# Tumor-Targeted and Biocompatible MoSe_2_ Nanodots@Albumin Nanospheres as a Dual-Modality Therapy Agent for Synergistic Photothermal Radiotherapy

**DOI:** 10.1186/s11671-019-2896-z

**Published:** 2019-02-26

**Authors:** Feng Qi, Ruizhen Liu

**Affiliations:** Department of Radiotherapy, First People’s Hospital of Shangqiu City, Shangqiu, 476100 China

**Keywords:** MoSe_2_, Biocompatibility, Photothermal therapy, Radiotherapy, Dual-modality therapy

## Abstract

Integrating multiple tumor therapy functions into one nanoplatform has been a new tumor therapy strategy in recent years. Herein, a dual-modality therapy agent consisting of molybdenum selenide nanodots (MoSe_2_ NDs) and bovine serum albumin (BSA) assembled nanospheres (MoSe_2_@BSA NSs) was successfully synthesized. After conjugation of folic acid (FA) molecules via polyethylene glycol (PEG) “bridges,” the FA-MoSe_2_@BSA NSs were equipped with tumor-targeting function. The BSA and PEG modifications provided the unstable MoSe_2_ NDs with excellent physiological stability. Since the end-product FA-MoSe_2_@BSA NSs had strong near-infrared (NIR) and X-ray absorbance properties, they exhibited good photothermal properties with excellent photothermal stability and radio-sensitization ability, hence, were explored as photothermal radiotherapy agents. In vitro and in vivo experiments indicated that the FA-MoSe_2_@BSA NSs possessed highly efficient tumor-targeting effect, great biocompability, and synergistic photothermal radiotherapy effect. This work suggests that such biocompatible FA-MoSe_2_@BSA NSs may be a promising multifunctional dual-modality tumor therapy agent for use in combination tumor therapy.

**Electronic supplementary material:**

The online version of this article (10.1186/s11671-019-2896-z) contains supplementary material, which is available to authorized users.

## Background

Globally, breast cancer occurs at very high incidence rate in women and is notorious for its low survival rate and high rates of metastasis and relapse [1–3]. Surgical resection, radiotherapy (RT), and chemotherapy are commonly used treatment strategies in practice even though these therapies have drawbacks [4]. RT is a highly effective therapy but also harmful to normal tissue. RT has been explored for enhanced therapeutic effect while decreasing its harmful effects. RT employs ionizing radiation (e.g., γ-ray, X-ray) to ionize water molecules into reactive free radicals that damage DNA in cancer cells locally, even in deep region [5]. Since tumor microenvironment was reported to be hypoxic, this was considered as one of the major obstacles for RT [6, 7]. Given these disadvantages, combining RT with other modality therapeutic strategies were reported to be efficient in augmenting the therapeutic effects. To date, photothermal therapy (PTT) has been explored extensively as a minimally invasive cancer treatment due to fewer side effects, high specificity, and minimal side effect to normal tissues [8–15]. However, PTT alone is often insufficient, due to incomplete tumor suppression, especially for the inaccessible tumors, which could potentially cause a tumor relapse [16–18]. Interestingly, PTT was reported to lead to near-infrared (NIR)-induced hyperthermia increasing intratumor blood circulation, and subsequently increased oxygen availability in the tumor microenvironment, causing the cells to be more sensitive to RT [19–21]. Combining RT with PTT could combine the advantages of both which is preferable for improving the therapeutic outcomes of cancer.

Recently, two-dimensional (2D)-layered transition metal dichalcogenides (TMDs), such as MoS_2_, WS_2_, ReS_2_, and so on, have been employed as the NIR adsorbing agents or radio-sensitizers for enhancing the efficacy of PTT or RT due to their physical properties [7, 19, 21]. Shen and co-authors have reported a bottom-up preparation of uniform ultrathin ReS_2_ nanosheets for image-guided highly effective PTT and RT [21]. In addition to these TMDs, molybdenum selenide (MoSe_2_) have been reported as a NIR photothermal transducer for PTT [22, 23]. Since PTT alone have its drawback, there is more reason for the exploitation of MoSe_2_ properties like radio-sensitization for better cancer therapy.

In this work, we first prepared the ultra-small MoSe_2_ nanodots, which were then assembled with bovine serum albumin (BSA) into nanospheres (NSs) and finally conjugated with tumor-targeting molecule folic acid (FA) via polyethylene glycol (PEG) “bridges.” In addition to the great photothermal effect, the obtained FA-MoSe_2_@BSA NSs were found to have excellent radio-sensitization property. The BSA modification endowed the MoSe_2_ nanodots (NDs) with excellent physiological stability and biocompability. In vitro and in vivo experiments demonstrated that the FA-MoSe_2_@BSA NSs exhibited excellent tumor-targeting effect, while simultaneously functioned as NIR photothermal agent and radio-sensitizer for synergistic photothermal radiotherapy with no toxicity to health tissue.

## Methods

### Materials

FA-PEG_5000_-NHS and CH_3_-PEG_5000_-NHS were obtained from Shanghai Ponsure Biotech. Co. Ltd. Fluorescein isothiocyanate (FITC), bovine serum albumin (BSA, purified ≥ 98.0%), bulk MoSe_2_ powder, and Calcein-AM (CA)-propidium iodide (PI) stain were purchased from Sigma-Aldrich (St. Louis, Mo, USA). 4′,6-Diamidino-2-phenylindole (DAPI) were obtained from Aladdin (Shanghai, China). Cell culturing reagents were all provided by Corning Inc. Cell counting kit-8 (CCK-8) was supplied by Dojindo Laboratories (Japan). The γ-H2AX antibody was supplied by Millipore (Temecula, CA).

### Preparation of FA-MoSe_2_@BSA NSs

Firstly, in a typical procedure, 50 mg MoSe_2_ powder was added into the 25 mL distilled water with 20-min stirring and then was sonicated in an ice-bath by using tip sonication (Scientz-IID, 950 W, 25 kHz). The sonication was pulsed for 2 s on and 3 s off for a total sonication time of 12 h with 70% amplitude. After that, the mixture was centrifuged with 6000 rpm for 25 min. The supernatant was collected and centrifuged again at 12,000 rpm for 30 min, resulting in MoSe_2_ nanodots (MoSe_2_ NDs) solution. Afterwards, 25 mg BSA powder were added into the above supernatant with slighted stirring, forming hardened coacervates after stirring for 6 h under 25 °C and pH = 7.4, and then was processed by cross-linking with 0.5% glutaraldehyde (250 μL). Afterwards, the glutaraldehyde was removed by dialyzing in water for 1 day, resulting in the MoSe_2_@BSA nanospheres (MoSe_2_@BSA NSs). Next, MoSe_2_@BSA nanospheres were divided into two parts, the one is mixed with FA-PEG_5000_-NHS (8 mg), and the other one was mixed with CH_3_-PEG_5000_-NHS (8 mg) solution and stirred for 2 h. At last, the solution was dialyzed in water to result in purified FA-MoSe_2_@BSA NSs and MoSe_2_@BSA NSs solution. The prepared solution was stored at 4 °C. In addition, UV-VIS spectrometer was used to quantify the FA on the FA-MoSe_2_@BSA NSs. In detail, after mixing MoSe_2_@BSA nanospheres with FA-PEG_5000_-NHS (8 mg) and reacting for 2 h, the mixture was centrifuged to remove the unbounded FA. The supernate was collected for absorption detection. The FA concentration was detected by a UV–vis spectrometer at FA absorption peak wavelength (280 nm). The FA encapsulation efficiency (EE) was calculated as described in the following equation:

$$ \mathrm{EE}\ \left(\%\right)=\frac{{\mathrm{FA}}_{\mathrm{total}}-{\mathrm{FA}}_{\mathrm{unloaded}}}{{\mathrm{FA}}_{\mathrm{total}}}\times 100\% $$

### Characterizations of FA-MoSe_2_@BSA NSs

The morphology of the samples was observed by transmission electron microscope (TEM, JEOL JEM2011, Tokyo, Japan) and scanning electron microscope (SEM, Hitachi FE-SEM S-9300, Janpan). Zetasizer (Nano ZS, Malvern Instruments Ltd., UK) was used to the size and zeta potential. The ultraviolet-visible (UV-Vis) absorption spectra were recorded on a UV2550 ultraviolet-visible spectrophotometer (Shimadzu, Kyoto, Japan). The Fourier transform infrared (FTIR) spectra was detected by a FTIR spectrometer (BRUKER VERTEX 70, Ettlingen, Germany). The X-ray powder diffraction (XRD) of the nanospheres was recorded by an X-ray diffractometer (Seifert Jso-Debyerex-2002, Germany). The content of Mo in cells and tissue was measured by inductively coupled plasma-atomic emission spectrometry (ICP-AES, Hitachi P4010, Japan). During NIR irradiation, variation of the temperature was recorded every 30 s using a thermocouple thermometer (Fluke, USA).

### Cell Culture and Cellular Uptake

Mouse mammary tumor 4T1 cells were purchased from the American Type Culture Collection (ATCC) and cultured in DMEM media supplemented with 10% FBS and 1% penicillin–streptomycin at 37 °C with 5% CO_2_.

For cellular uptake, FITC was used to label the NSs through physical absorption. 4T1 cells adhered to glass slides in 6-well plates and were incubated with free FITC, MoSe_2_@BSA NSs, FA-MoSe_2_@BSA NSs + FA, and FA-MoSe_2_@BSA NSs at the same concentration of FITC (0.05 mg/mL) for 3 h, respectively. The cells were then washed with PBS thrice and fixed by 0.2 mL of glutaraldehyde, followed by staining with DAPI for 10 min. The fluorescence images of cells were captured using the laser scanning microscope. To further observe the cellular uptake, 4T1 cells (2 × 10^5^ cells/well) were cultured in 6-well cell culture plate for 24 h and then incubated with MoSe_2_@BSA NSs, FA-MoSe_2_@BSA NSs + FA, and FA-MoSe_2_@BSA NSs for extra 3 h. After that, the treated cells were gently washed by PBS for three times, homogenized, and treated with 1 mL aqua regia solution for 4 h. ICP-AES was used to detect the Mo content inside the cells. Uptake ratio = $\frac{\mathrm{Ma}}{\mathrm{Mb}}$× 100%, where Ma is the mass of the Mo inside cells, and Mb is the mass of Mo total added.

### In Vitro Biocompatibility

Firstly, health mouse whole blood was collected to detect the in vitro hemolysis of the FA-MoSe_2_@BSA NSs. In detail, red blood cells (RBCs) were collected by centrifugation. Discarding the supernatants, the collected RBCs were mixed with FA-MoSe_2_@BSA NSs (in PBS, 1:4) at predetermined concentrations (50 μg/mL, 100 μg/mL, 150 μg/mL, 200 μg/mL, and 400 μg/mL). As positive or negative control, RBCs were incubated with deionized water or PBS. After standing incubated at 37 °C for 1 h, the above set of suspensions were centrifuged (10,000 rpm, 1 min) and the absorbance of the supernatants at 541 nm was monitored by a UV-Vis spectrometer. The hemolysis ratio was calculated using the following equation.

$$ \mathrm{HR}\ \left(\%\right)=\frac{\ {A}_t-{A}_{nc}}{A_{pc}-{A}_{nc}}\times 100\% $$

where *A*_*t*_, *A*_*pc*_, and *A*_*nc*_ are the absorbance of the supernatant at 541 nm of the test sample, positive and negative controls, respectively.

In addition, the cytotoxicity of MoSe_2_@BSA NSs and FA-MoSe_2_@BSA NSs was detected by a standard CCK-8 assay. 4T1 cells (1 × 10^5^ cells/mL, 0.5 mL) were seeded in 96-well plate and cultured for 24 h. After discarding the old media, fresh media containing 0.01, 0.1, 0.15, 0.3, and 0.4 mg/mL of MoSe_2_@BSA NSs and FA-MoSe_2_@BSA NSs were incubated with 4T1 cells for 24 h. PBS was used to mildly wash the cells three times. A 100 μL CCK-8 working solution (10% CCK-8 + 90% DMEM) was then added to each well, followed by incubation at 37 °C for 1 h. The absorbance value at 450 nm was detected using a microplate reader (Labtech, Inc., Durham, North Carolina).

### In Vitro Photothermal Radiotherapy

Firstly, the in vitro photothermal performance of the NSs was investigated. MoSe_2_@BSA NSs and FA-MoSe_2_@BSA NSs with the same Mo concentrations cultured with cells for 3 h and then irradiated by NIR irradiation for 5 min (808 nm, 1 W/cm^2^). The temperature of the treated cells in each well were detected with an infrared thermal camera (Fluke TI25, USA), respectively.

Next, for in vitro photothermal therapy, adherent 4T1 cells were cultured with different concentration of MoSe_2_@BSA NSs and FA-MoSe_2_@BSA NSs for 3 h. The NSs outside the cells were removed. The cells were then treated with or without NIR (808 nm, 1 W/cm^2^, 5 min) and different dosage of X-ray irradiation (RT, 0–5 Gy, 0.084 Gy/s). After another 24-h incubation, cell viability was detected by a standard CCK-8 assay. The treated cells above were further co-stained by calcein-AM/PI to detect the live and dead cells and then imaged by a confocal laser scanning microscope (calcein-AM: Ex = 488 nm, Em = 515 nm; PI: Ex = 535 nm, Em = 617 nm). Moreover, the treated cells were also analyzed by γ-H2AX immunofluorescence. After the treatment above, the cells were fixed by 4% paraformaldehyde for 10 min and permeabilized with methanol for 15 min at − 20 °C and washed with PBS. Afterwards, the cells were mixed with a blocking buffer (1% BSA in PBS solution) for 1 h at 25 °C and further incubated with anti-phospho-histone γ-H2AX mouse monoclonal antibody (dilution 1:500) overnight at 4 °C. After PBS washing, the fluorescence of the cells was observed by confocal laser scanning microscope.

### Animal Model

Balb/c nude mice (5–8-week-old) were provided from Charles River Laboratories (Beijing, China). To establish animal 4T1 tumor model, 150 μL of 10^6^ suspension cells were subcutaneous injected into the back of mouse. The mice were fed in animal room and observed every 2 days. All welfare and experimental procedures in this study were performed in accordance with the policies of National Ministry of Health and approved by the Ethics Committee of the First People’s Hospital of Shangqiu City. When the tumor volume reached 100 mm^3^, the mice were applied for in vivo experiments.

### In Vivo Biodistribution and Blood Circulation

Systemic biodistribution of the NSs was investigated in 4T1 tumor-bearing mice. At 1 h, 1 day, 7 days, and 24 days post intravenous injection of MoSe_2_@BSA NSs and FA-MoSe_2_@BSA (10 mg/kg), the tumor and major organs (heart, liver, spleen, lung, and kidney) were weighed and digested by aqua regia solution 12 h. The Mo and Se content in these tissues was analyzed by an ICP-AES. In addition, healthy Balb/c mice were intravenously injected with FA-MoSe_2_@BSA (10 mg/kg). Approximately 10 μL of blood from the tail of mice was collected and analyzed by an ICP-AES for blood circulation.

### In Vivo Photothermal Radiotherapy

For in vivo photothermal radiotherapy, tumor-bearing mice (*n* = 5 per group) were treated with PBS + NIR, PBS + RT, MoSe_2_@BSA NSs + NIR + RT, FA-MoSe_2_@BSA NSs + RT, FA-MoSe_2_@BSA NSs + NIR, and FA-MoSe_2_@BSA NSs + NIR + RT (with 5 mg/kg of MoSe_2_). The radiotherapy dose was 5 Gy. At 24-h intravenous post injection, tumor region was irradiated by 5 min NIR irradiation (808 nm, 1 W/cm^2^). During the irradiation, the thermal images of the mice were recorded by infrared thermal camera. In the following 30 days, the length and width of the tumor were monitored every 4 days. The relative tumor volume was calculated as *V*/*V*_0_, where *V*_0_ represents tumor volume when the treatment was initiated. Meanwhile, the body weight of each mouse was also monitored every 4 days.

### In Vivo Biocompatibility

For in vivo biocompatibility, 150 μL of FA-MoSe_2_@BSA NSs (15 mg/kg) was intravenously injected into healthy Balb/c nude mice. Before injection and after 30 days, the blood were collected for complete blood counts evaluations including white blood cell (WBC), red blood cells (RBC), hemoglobin (HGB), mean platelet volume (MPV), mean corpuscular hemoglobin (MCH), hematocrit (HCT), mean corpuscular hemoglobin concentration (MCHC), mean corpuscular volume (MCV), and platelet (PLT). At the same time, the mice were sacrificed and the heart, liver, spleen, lung, and kidney were collected. The obtained organs were fixed with 4% paraformaldehyde and sectioned into 5 μm slices and stained with hematoxylin and eosin (H&E). The stained sections were imaged by a digital microscope.

### Statistical Analysis

Data were shown as mean ± SD. Two-tailed Student’s *t* test was used to analyze the statistical significance of two groups. The differences were considered significant for *P < 0.05 and highly significant for **P < 0.01.

## Results and Discussion

### Preparation and Characterization of FA-MoSe_2_@BSA NSs

**Fig. 1:**
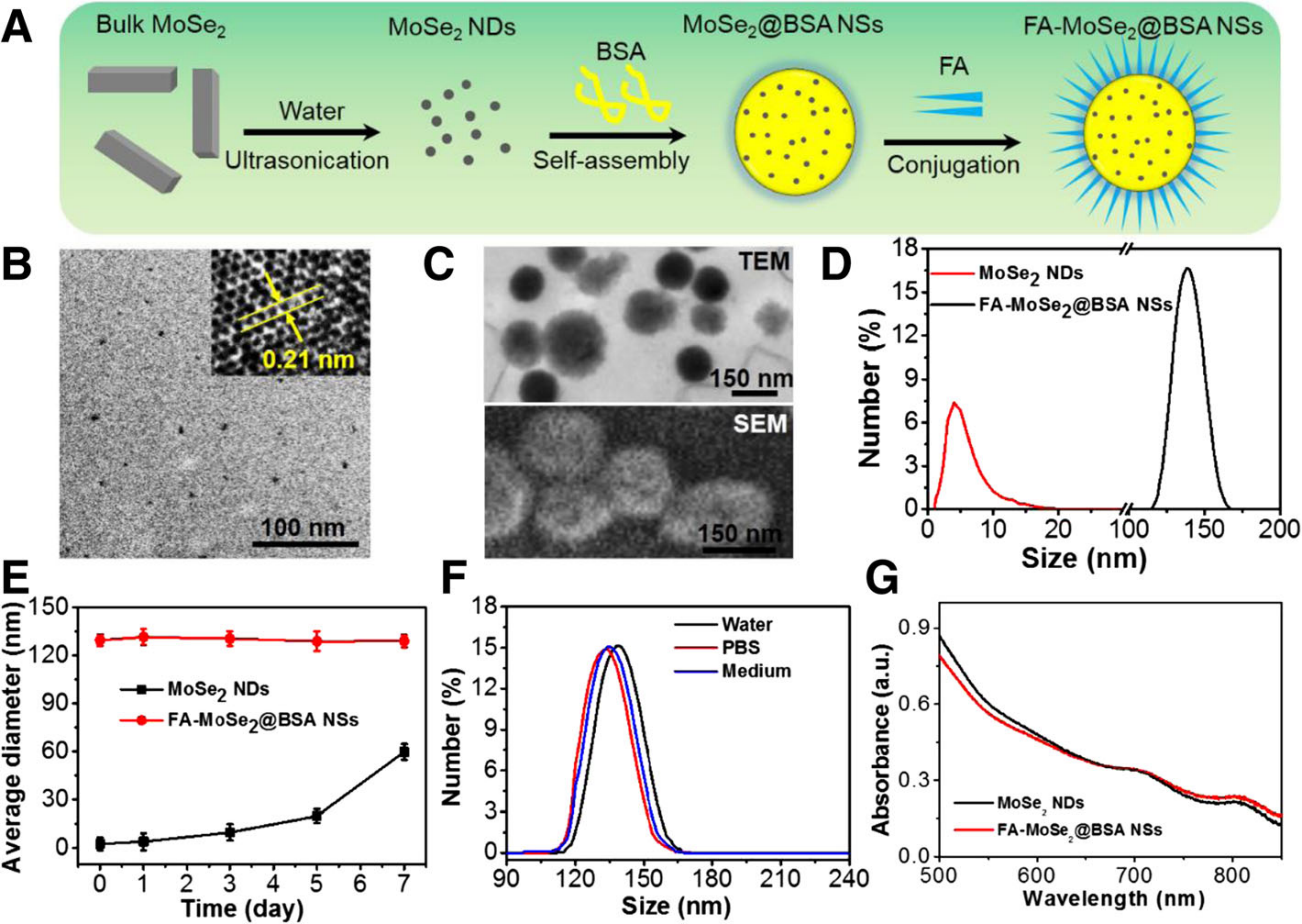
**a** A schematic of FA-MoSe_2_@BSA NSs synthesis*.* **b** TEM image of MoSe_2_ NDs. Inset was the high-resolution TEM image. **c** The TEM and SEM images of FA-MoSe_2_@BSA NSs. **d** The size distribution of MoSe_2_ NDs and FA-MoSe_2_@BSA NSs. **e** The size change of MoSe_2_ NDs and FA-MoSe_2_@BSA NSs in water over 7 days. **f** The size distribution of FA-MoSe_2_@BSA NSs in water, PBS, and medium, respectively. **g** The absorbance spectra of MoSe_2_ NDs and FA-MoSe_2_@BSA NSs

The preparation procedure of the FA-MoSe_2_@BSA NSs is depicted in Fig. 1a. Briefly, unstable MoSe_2_ nanodots were prepared from bulk MoSe_2_ under ultrasonication, then stabilized and assembled by BSA protein, and conjugated simultaneously to the target molecule FA via PEG “bridges.” The prepared MoSe_2_ NDs were ultra-small nanodots as observed in TEM (Fig. 1b). The XRD pattern of MoSe_2_ NDs was shown in Additional file 1: Figure S1. The diffraction peak at 13.1° belongs to the (002) plane, matching the peak position of bulk MoSe_2_. The distinct (002) peak indicates the existence of few-layers in the c-axis of MoSe_2_ NDs. The diffraction peak of (100) plane for MoSe_2_ NDs broadens significantly in comparison with bulk MoSe_2_, which may originate from the size reduction of MoSe_2_ NDs [23]. Following assembly and conjugation with BSA protein and FA, the nanocomposites formed sphere-like particles (Fig. 1c). The FTIR spectra showed the existence of –CONH- bond in FA-MoSe_2_@BSA NSs, indicating that FA was likely conjugated onto MoSe_2_ through ester bond (Additional file 1: Figure S2). In addition, we have quantified the FA on the FA-MoSe_2_@BSA nanospheres, which was 10.5 ± 0.11%. DLS analysis revealed that the average diameters of MoSe_2_ NDs and FA-MoSe_2_@BSA NSs were approximately 3.8 nm and 139.8 nm, respectively (Fig. 1d). After 7 days of storage, the size of MoSe_2_ NDs increased from 3.8 to 63.2 nm, while FA-MoSe_2_@BSA NSs showed no obvious change in size (Fig. 1e), indicating aggregation and the low stability of MoSe_2_ NDs in long-term storage. In addition, when dispersed in different media like water, PBS, and cell medium, FA-MoSe_2_@BSA NSs displayed similar size distribution (Fig. 1f). These results indicated the FA-MoSe_2_@BSA NSs are stable in physiological conditions and this improved stability of MoSe_2_ NDs is likely attributed to BSA assembly and PEG coating [24, 25]. As shown in Fig. 1g, the ultraviolet-visible (UV-Vis) spectra of MoSe_2_ NDs and FA-MoSe_2_@BSA NSs had similarly high NIR absorbance characteristics, indicating that the BSA and FA modifications did not affect the absorbance of MoSe_2_.

### Photothermal Effect of FA-MoSe_2_@BSA NSs

**Fig. 2:**
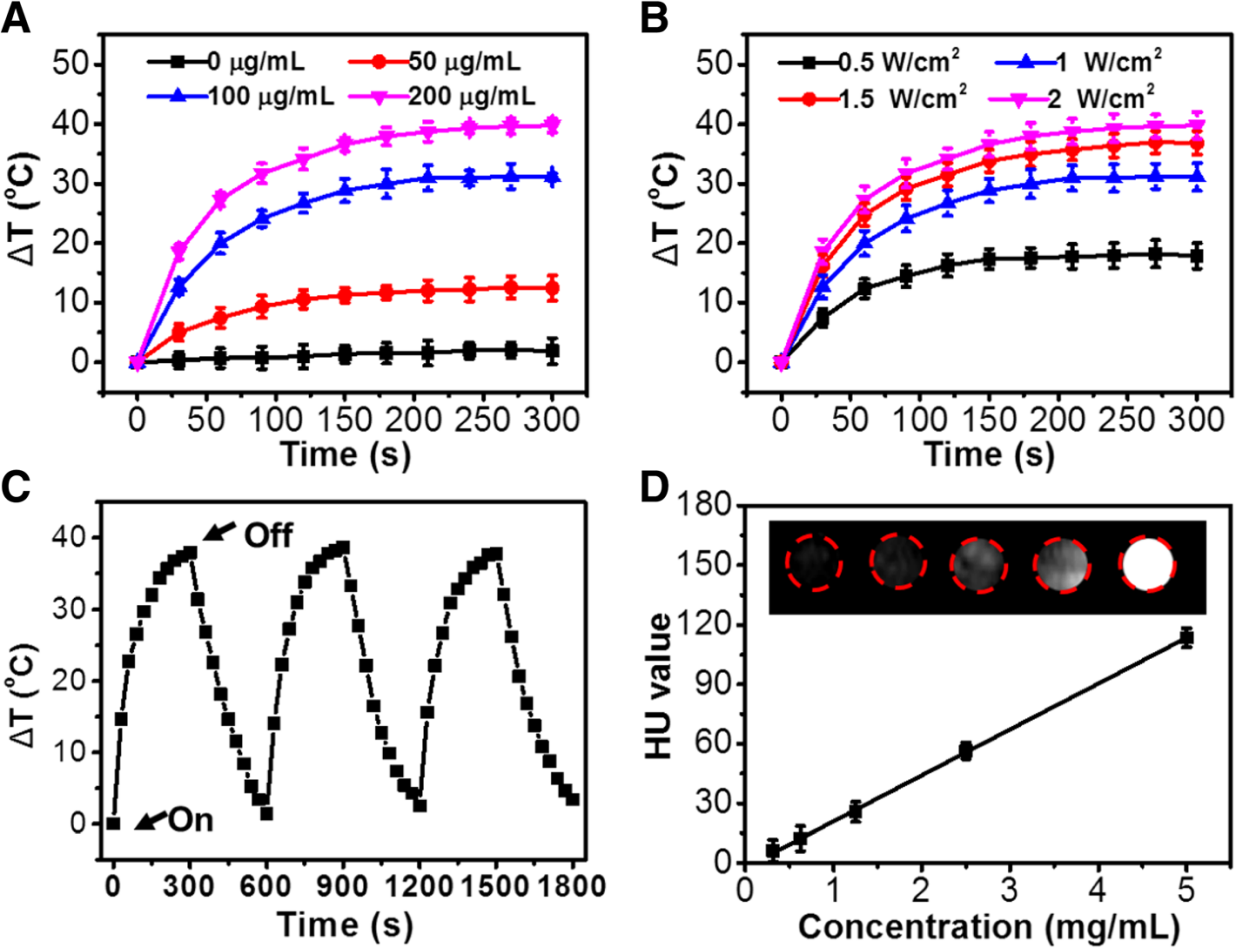
**a** Photothermal heating curves of FA-MoSe_2_@BSA NSs solution at different concentrations (0, 50, 100, and 200 μg/mL) under 808 nm laser irradiation at the power density of 1 W/cm^2^. **b** Photothermal heating curves of FA-MoSe_2_@BSA NSs solution at 100 μg/mL under 808 nm laser irradiation at different power density (0.5, 1, 1.5, and 2 W/cm^2^). **c** Temperature variations of FA-MoSe_2_@BSA NSs under three cycles of 808 nm laser irradiation at the power density of 1 W/cm^2^. **d** CT images (inset) and HU values of FA-MoSe_2_@BSA NSs with different concentrations

Figure 2a shows that the temperature of FA-MoSe_2_@BSA NSs solution increased with increasing concentrations (0–200 μg/mL). After NIR irradiation (808 nm, 1 W/cm^2^) for 5 min, the temperature change of FA-MoSe_2_@BSA NSs solution at 200 μg/mL reached approximately 41 °C, while the temperature of pure water only increased by approximately 1.5 °C under the same conditions. Moreover, the temperature change of FA-MoSe_2_@BSA NSs irradiated under different power intensity (0.5–2.0 W/cm^2^) for 5 min was also recorded. As shown in Fig. 2b, the temperature increased to a maximum of 40.6 °C with increasing laser power intensity. Figure 2c depicts the photostability of FA-MoSe_2_@BSA NSs, implying that the FA-MoSe_2_@BSA NSs retained its excellent photothermal effects without any attenuation of the temperature elevation after three cycles of NIR irradiation. These results demonstrated that FA-MoSe_2_@BSA NSs had significant photostability and excellent photothermal properties. As shown in Fig. 2d, the Hounsfield unit (HU) values of FA-MoSe_2_@BSA NSs images obtained with computed tomography (CT) were positively correlated with their concentrations, indicating the NSs could potentially be used as a radio-sensitizer.

### Cellular Uptake and In Vitro Biocompatibility

**Fig. 3:**
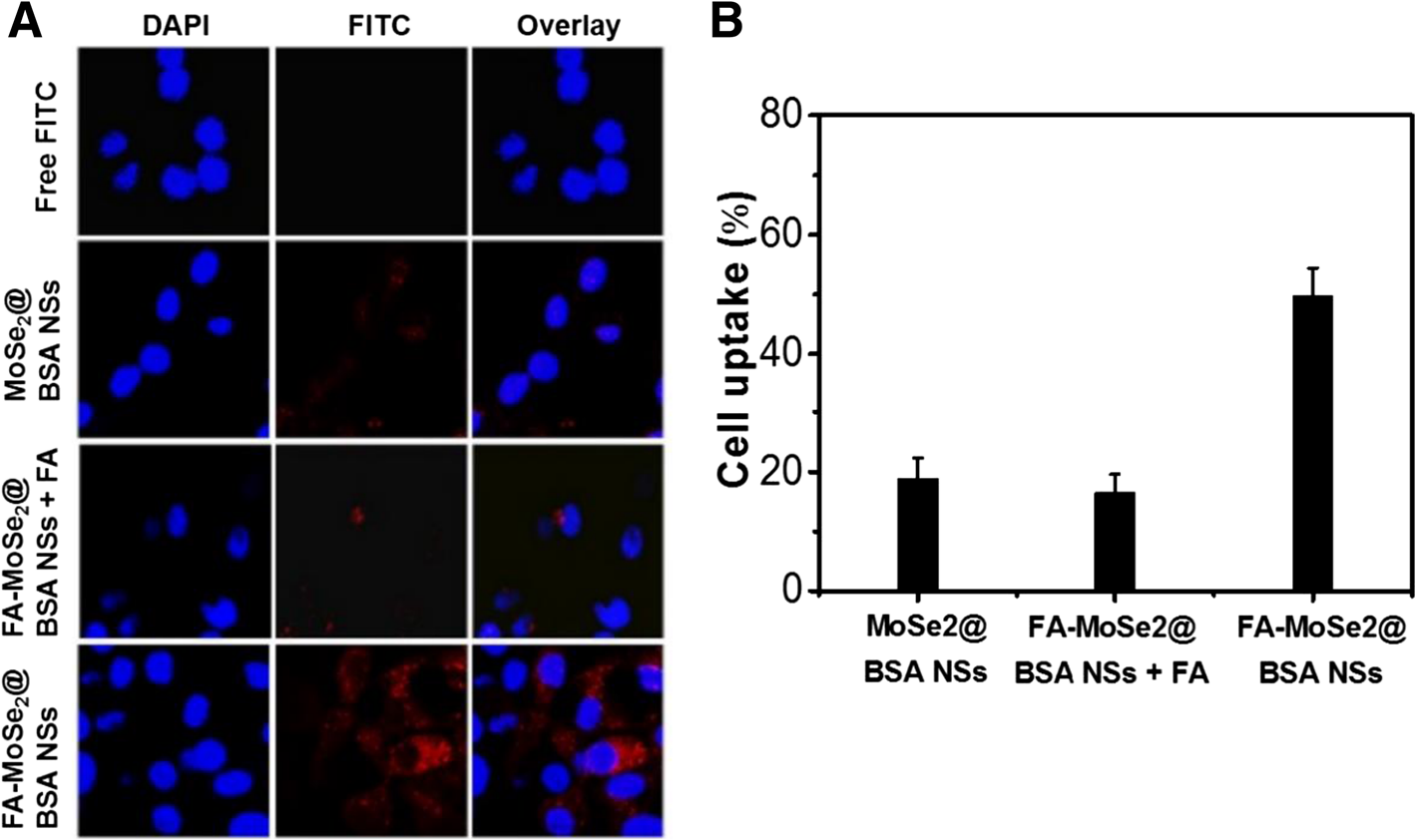
**a** Confocal fluorescence images of 4T1 cells after incubation with free FITC, and FITC-labeled MoSe_2_@BSA NSs, FA-MoSe_2_@BSA NSs + FA blocking, and FA-MoSe_2_@BSA NSs. Red and blue colors represent FITC fluorescence and DAPI stained cell nuclei, respectively. **b** ICP-AES quantitative analysis of 4T1 cells toward MoSe_2_@BSA NSs, FA-MoSe_2_@BSA NSs + FA blocking, and FA-MoSe_2_@BSA NSs

To evaluate the cellular uptake, MoSe_2_@BSA NSs and FA-MoSe_2_@BSA NSs were labeled by FITC. As depicted in Fig. 3a, much stronger FITC fluorescence was observed inside the cytoplasm in FA-MoSe_2_@BSA NSs-treated cells, compared to that of MoSe_2_@BSA NSs- and free FITC-treated cells. ICP-AES quantitative analysis showed higher cellular uptake of FA-MoSe_2_@BSA NSs than MoSe_2_@BSA NSs (Fig. 3b). These results demonstrated that FA enhanced the cellular uptake of FA-MoSe_2_@BSA NSs. Interestingly, after an FA blocking, the FA-MoSe_2_@BSA NSs-treated cells showed weaker green FITC fluorescence inside the cytoplasm compared with that of without FA blocking. Corresponding, the cell uptake rate of FA-MoSe_2_@BSA NSs + FA-treated cells is less than that of FA-MoSe_2_@BSA NSs-treated cells. It indicates that the FA receptor on the cell membrane is hindered (by free FA), in turn, reduces the targeting ability and accessibility of FA-MoSe_2_@BSA NSs. It further demonstrates that FA receptor is over expressed in 4T1 cells and FA-MoSe2@BSA NSs enter cells probably through a receptor-mediated endocytosis pathway [26, 27].

**Fig. 4:**
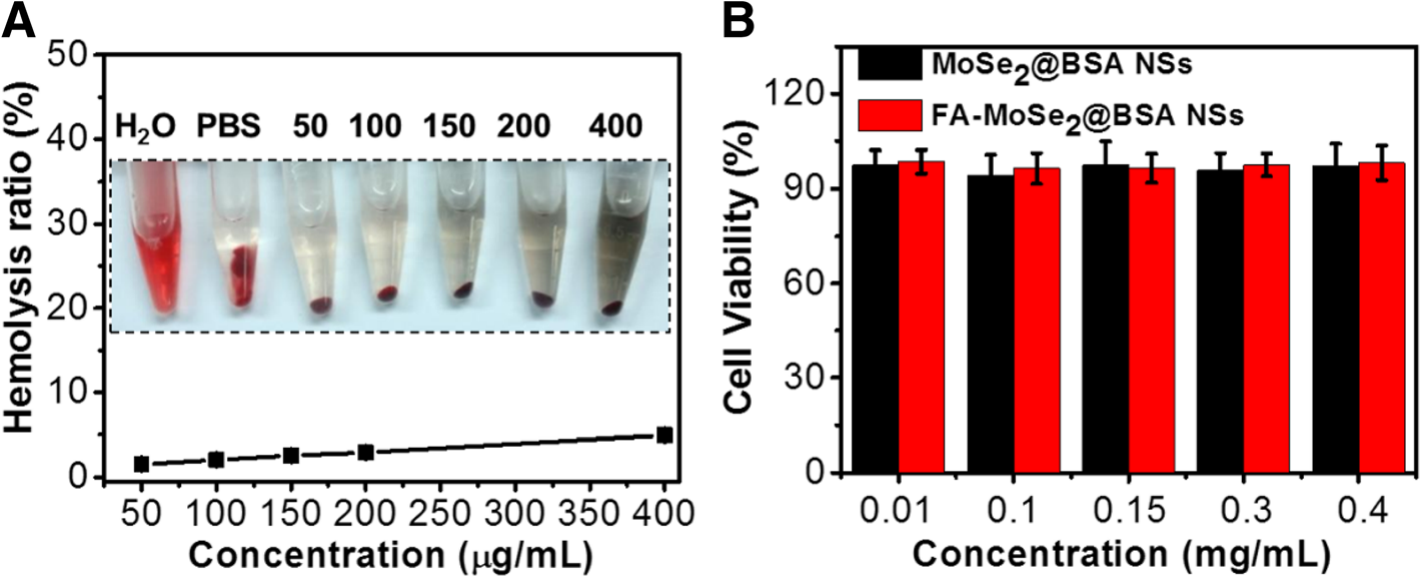
**a** Hemolysis ratio of RBCs after 1-h incubation with FA-MoSe_2_@BSA NSs at different concentrations. The inset shows the photograph of RBCs exposed to distilled water, PBS, and FA-MoSe_2_@BSA NSs with different concentrations followed by centrifugation. **b** Cell viability of 4T1 cells after treatment with towards MoSe_2_@BSA NSs and FA-MoSe_2_@BSA NSs at different concentrations for 24 h

The in vitro biocompability of the NSs was evaluated by hemolysis and cytotoxicity analyses. Figure 4a showed no obvious hemolysis for FA-MoSe_2_@BSA NSs-treated red blood cells (RBCs) or PBS-treated RBCs. Moreover, when concentrations of up to 0.4 mg/mL of MoSe_2_@BSA NSs and FA-MoSe_2_@BSA NSs were incubated with cells for 24 h, there were less than 10% viability suppression. These results suggested that the FA-MoSe_2_@BSA NSs have great in vitro biocompatibility.

### In Vitro Photothermal Radiotherapy

**Fig. 5:**
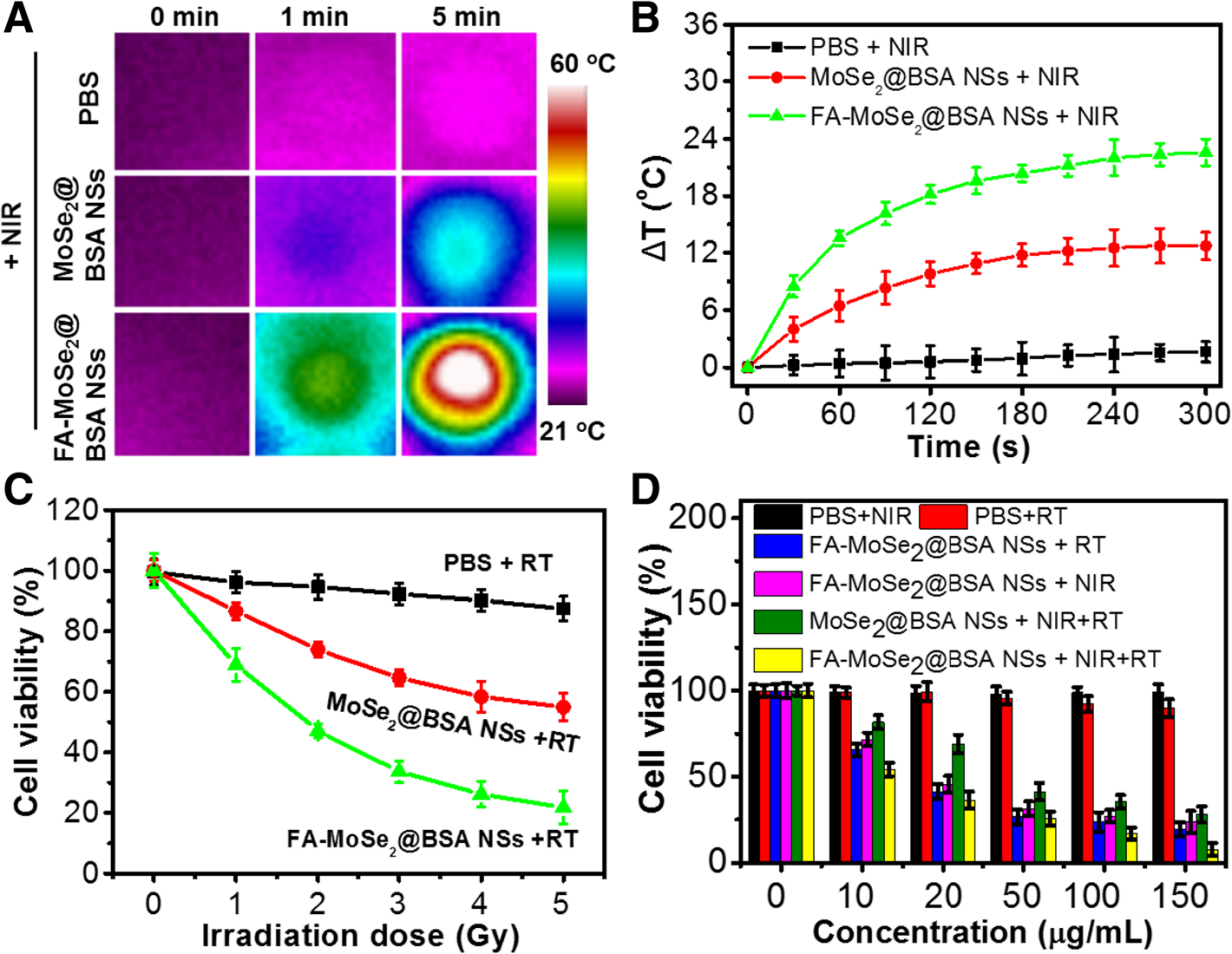
**a** Thermal images of PBS, MoSe_2_@BSA NSs and FA-MoSe_2_@BSA NSs treated cells, and **b** the corresponding temperature change curves under continuous irradiation with an 808 nm laser (1 W/cm^2^). **c** Cell viability of 4 T1 cells treated with PBS, MoSe_2_@BSA NSs, and FA-MoSe_2_@BSA NSs combined with different dose of X-ray irradiation (0–5 Gy). **d** Cell viability of 4T1 cells treated with different samples under 808 nm NIR laser (5 min, 1 W/cm^2^) and X-ray (5 Gy) irradiation

As shown in Fig. 5a, b, cells treated with FA-MoSe_2_@BSA NSs showed the highest temperature increase (ΔT = 23.6 °C) after 5 min of NIR irradiation (808 nm, 1 W/cm^2^) compared to MoSe_2_@BSA NSs- and PBS-treated cells. Figure 5c showed an enhancement of RT efficacy by addition of FA-MoSe_2_@BSA NSs. With increasing X-ray doses, the RT efficacy of FA-MoSe_2_@BSA NSs improved much more than that of MoSe_2_@BSA NSs. It was demonstrated that FA-MoSe_2_@BSA NSs could enhance radiotherapy effect, probably due to their X-ray attenuation capability that could concentrate X-ray radiation energy inside tumor cells and generate secondary Auger electrons, resulting in DNA damage and suppression of cell growth [28, 29].

For further evaluation of combined RT and PTT therapeutic effect, 4T1 cells were treated with only NIR or RT, FA-MoSe_2_@BSA NSs + RT, FA-MoSe_2_@BSA NSs + NIR, or FA-MoSe_2_@BSA NSs + NIR + RT. As shown in Fig. 5d, FA-MoSe_2_@BSA NSs + NIR + RT-treated group displayed the most significant concentration-dependent cell death, with 92.8% suppression rate. The excellent therapeutic effect of the FA-MoSe_2_@BSA NSs was likely due to (1) the photothermal ablation of PTT and DNA damage by RT of FA-MoSe_2_@BSA NSs, and (2) FA targeting enhanced the cell internalization of FA-MoSe_2_@BSA NSs and thus the generation of more heat and X-ray to kill the cells.

**Fig. 6:**
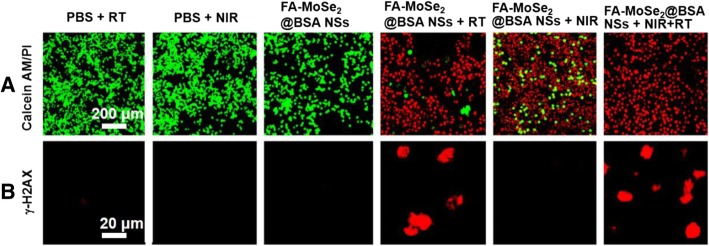
**a** Live−dead and **b** γ-H2AX staining images of 4T1 cells treated with PBS + RT, PBS + NIR, FA-MoSe_2_@BSA NSs, FA-MoSe_2_@BSA NSs + NIR, FA-MoSe_2_@BSA NSs + RT, and FA-MoSe_2_@BSA NSs + NIR + RT, respectively

As shown in Fig. 6a, few dead cells could be observed in the PBS + NIR and PBS + RT control groups. Even though dead cells were found in FA-MoSe_2_@BSA NSs + RT or FA-MoSe_2_@BSA NSs + NIR groups, but living cells still existed. Conversely, in the FA-MoSe_2_@BSA NSs + NIR + RT group, more than 95% of cells were damaged and showed red fluorescence, indicating that combined RT and PTT of FA-MoSe_2_@BSA NSs could enhance the therapeutic efficiency.

Since FA-MoSe_2_@BSA NSs have high X-ray absorbance, it would be potential to have the capability to enhance RT. As shown in Fig. 6b, very low levels of γ-H2AX signals were observed in the PBS-treated groups, FA-MoSe_2_@BSA NSs only, and FA-MoSe_2_@BSA NSs + NIR-treated groups. Meanwhile, FA-MoSe_2_@BSA NSs + NIR + RT-treated group showed higher levels of γ-H2AX signals, indicating more significant DNA damage inside cell nuclei. These results demonstrated that FA-MoSe_2_@BSA NSs could enhance RT effect attributing to its X-ray attenuation capability, which would concentrate X-ray radiation energy inside tumor cells and generate secondary and Auger electrons to cause DNA damages and suppression of cell growth [30–32].

### In Vivo Biodistribution and Blood Circulation

**Fig. 7:**
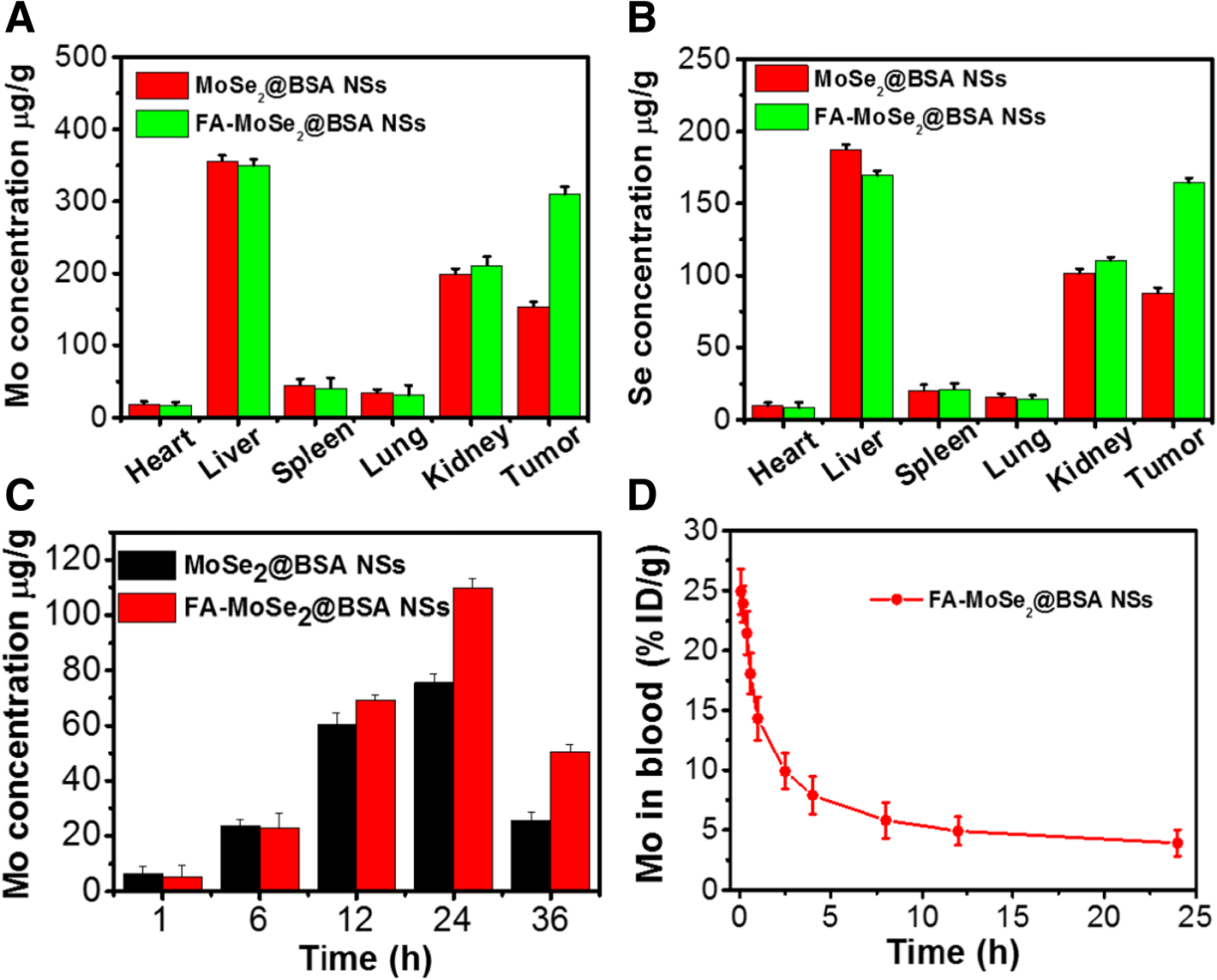
The **a** Mo and **b** Se elements content of tumor and major organs including heart, liver, spleen, lung, and kidney in MoSe_2_@BSA NSs and FA-MoSe_2_@BSA NSs-treated mice. **c** Quantitative in vivo analysis of the Mo elements content of the tumor regions in MoSe_2_@BSA NSs and FA-MoSe_2_@BSA NSs-treated mice as a function of injection time. **d** Blood circulation curve of Mo at different time points post-injection of FA-MoSe_2_@BSA NSs

As shown in Fig. 7a, b, at 24-h post-injection, the Mo and Se elements accumulated in the tumor except for liver and kidney. The contents of Mo elements in tumor tissue post-injections of MoSe_2_@BSA NSs and FA-MoSe_2_@BSA NSs were also evaluated. As shown in Fig. 7c, the Mo levels in tumor tissue for FA-MoSe_2_@BSA NSs-treated group increased with time and reached the peak at 24-h post-injection, which was higher than that of MoSe_2_@BSA NSs-treated group. Figure 7d shows the blood circulation curve of Mo at different time point post-injection of FA-MoSe_2_@BSA NSs. The Mo *t*_1/2_α (blood distribution half-life) and *t*_1/2_β (blood terminal elimination half-life) of the FA-MoSe_2_@BSA NSs group are 0.91 ± 0.06 h and 16.96 ± 1.3 h, respectively. These results were likely due to (1) prolonged blood circulation promoted by PEG and BSA modifications [24, 33], (2) decreased macrophage clearance of nanoparticles by the reticuloendothelial system [34, 35], and (3) facilitated tumor targeting effect by the FA modification and subsequent accumulation in tumor tissues. The tumor optimum accumulation time of FA-MoSe_2_@BSA NSs could guide the in vivo photothermal radiotherapy.

### In Vivo Photothermal Radiotherapy

**Fig. 8:**
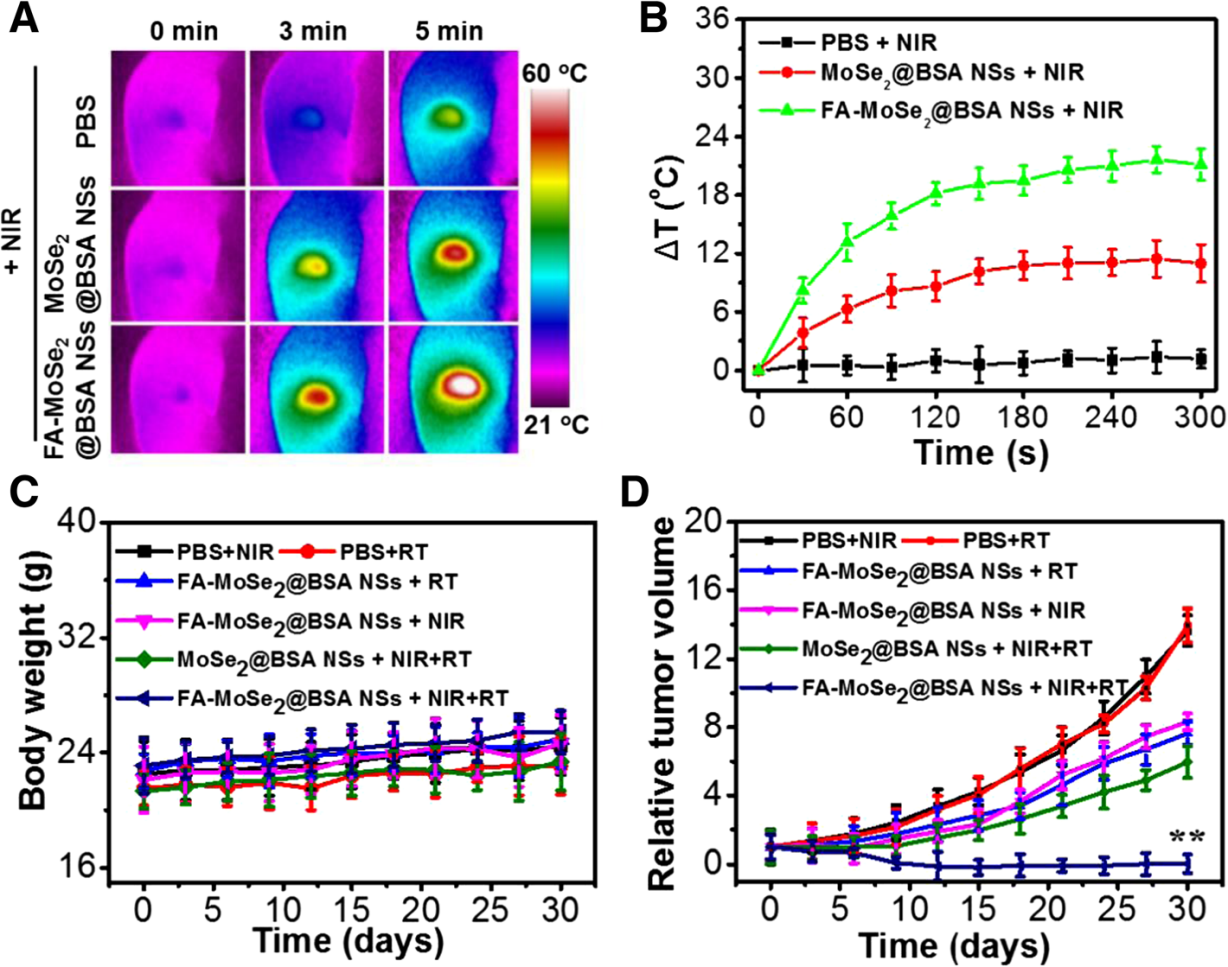
**a** In vivo thermal images of mouse after intravenous injection of saline, MoSe_2_@BSA NSs and FA-MoSe_2_@BSA NSs and durations NIR irradiation (808 nm, 1 W/cm^2^). **b** The corresponding temperature change curves of tumor regions in mice. **c** The weight and **d** relative tumor volume profile of 4T1 xenografted tumors after intravenous injection of PBS + RT, PBS + NIR, FA-MoSe_2_@BSA NSs + NIR, FA-MoSe_2_@BSA NSs + RT, MoSe_2_@BSA NSs + NIR + RT, and FA-MoSe_2_@BSA NSs + NIR + RT, respectively. **P < 0.01 vs control group and other groups

As shown in Fig. 8a, b, the temperature of the tumor region under NIR irradiation (5 min, 1 W/cm^2^) showed about 22.1 °C increase at 24-h post-injection of FA-MoSe_2_@BSA NSs, compared with that of PBS- or MoSe_2_@BSA NSs-treated groups. This indicated that FA-MoSe_2_@BSA NSs have excellent photothermal effect in vivo. As shown in Fig. 8c, no distinct weight changes were observed in the control or any of the treated Balb/c mice during the 30-day treatment duration, demonstrating that the treatments did not affect the health of these mice. Next, 4T1-tumor-bearing mice were randomly divided into six groups. Group 1: NIR; group 2: RT; group 3: FA-MoSe_2_@BSA NSs + RT; group 4: FA-MoSe_2_@BSA NSs + NIR; group 5: MoSe_2_@BSA NSs + NIR + RT; and group 6: FA-MoSe_2_@BSA NSs + NIR + RT. Then, 5 mg/kg of MoSe_2_ was used in all groups. The radiotherapy dose was 5 Gy, and the dose rate is 0.084 Gy/s. At 24-h intravenous post injection, tumor region was irradiated by 5 min NIR irradiation (808 nm, 1 W/cm^2^). The tumor sizes were closely monitored afterward (Fig. 8d). Compared to other groups, the most remarkable tumor growth inhibition was observed in group 6 after the combined photothermal-radiotherapy with FA-MoSe_2_@BSA NSs, achieving an obvious synergistic therapeutic outcome in comparison to PTT alone or RT alone delivered by FA-MoSe_2_@BSA NSs (Fig. 8d).

### In Vivo Biocompatibility

**Fig. 9:**
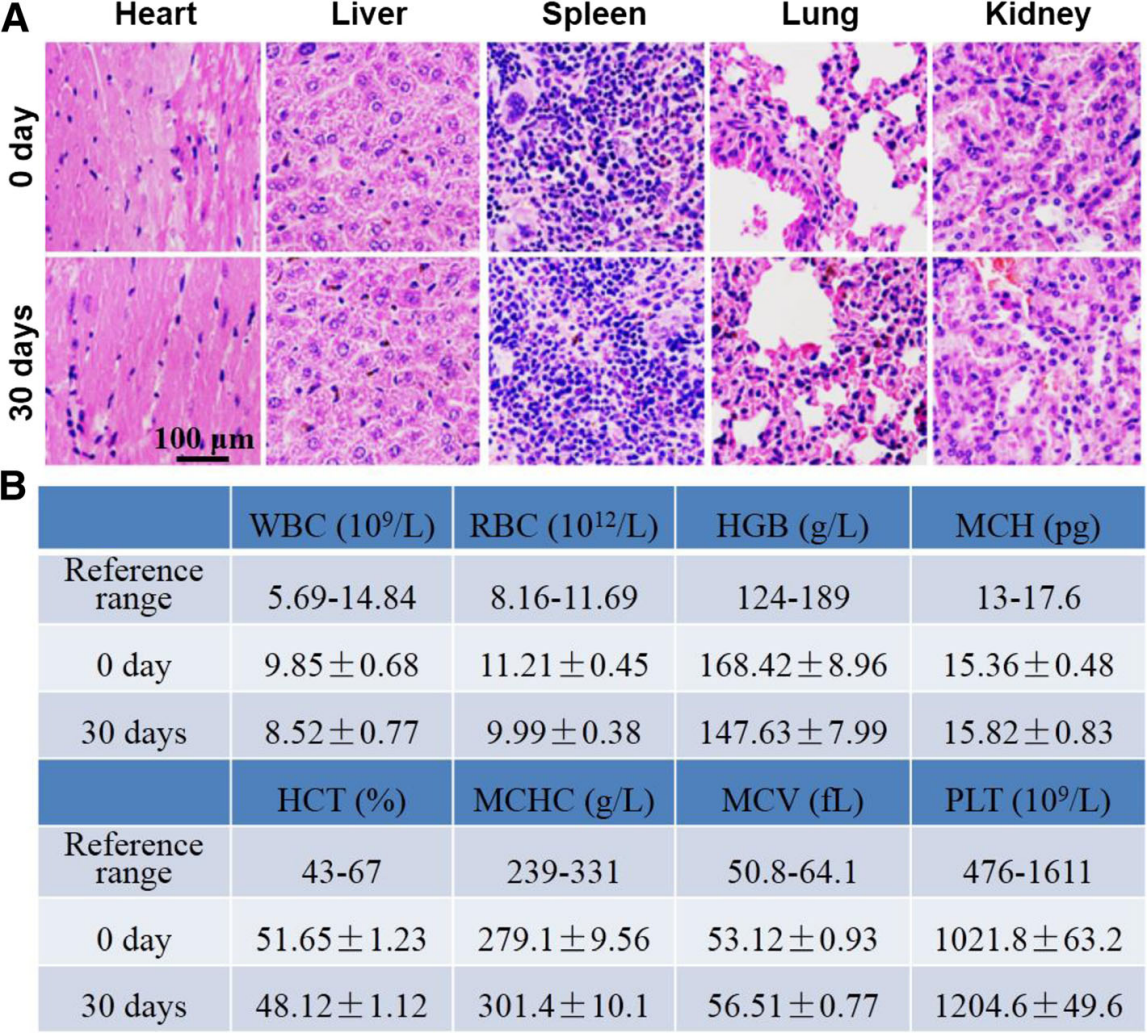
**a** H&E-stained tissue sections of major organs, including the heart, liver, spleen, lung, and kidney from mice treated with FA-MoSe_2_@BSA NSs at day 0 and day 30 (scale bar = 100 μm). **b** Blood biochemistry of mice at days 0 and 30 post-treatment with FA-MoSe_2_@BSA NSs

As a kind of nanoagent for in vivo biomedical applications, their potential toxic side effect is something that always requires particular attention. In addition to the body weight data of mice in different groups post various treatments in Fig. 8c, the H&E staining images of major organs and complete blood panel assays were provided to evaluate the safety of the FA-MoSe_2_@BSA NSs. As shown using H&E staining in Fig. 9a, no apparent pathological tissue damage or abnormality in major organs (heart, liver, spleen, lung, and kidney) was observed in FA-MoSe_2_@BSA NSs-treated mice. Moreover, as illustrated in Fig. 9b, the parameters of WBC, RBC, HGB, MCH, HCT, MCHC, MCV, and PLT for FA-MoSe_2_@BSA NSs-treated mice were within the normal range. These results demonstrated that FA-MoSe_2_@BSA NSs exhibited low toxicity and excellent in vivo biocompatibility.

## Conclusions

In summary, MoSe2 NDs was first prepared by ultrasonication, and MoSe_2_@BSA nanospheres was then successfully synthesized via a simple BSA self-assembly method. The BSA surface provided a rich modifiable functional group that readily conjugated FA molecules, enabling the synthesis of versatile FA-MoSe_2_@BSA NSs which showed outstanding physiological stability and excellent tumor targeting effect. Due to the strong radio-sensitization ability and high NIR absorption of MoSe_2_ NDs, FA-MoSe_2_@BSA NSs could be used as a photothermal agent for NIR-induced tumor ablation, and act as a radio-sensitizer to enhance the efficacy of RT. In vitro and in vivo experiments verified that FA-MoSe_2_@BSA NSs exhibited high cytotoxicity under NIR and X-ray irradiation, contributing to remarkably enhanced therapeutic effect in the tumor-targeted combined photothermal-radiotherapy. Most importantly, it was demonstrated that FA-MoSe_2_@BSA NSs have great biocompability in vitro and in vivo, encouraging further biomedical or clinic applications. Therefore, considering all the above desirable characteristics, the FA-MoSe_2_@BSA NSs with highly integrated functionalities is promising for applications in cancer therapy.

## Additional File

Additional file 1: **Figure S1.** XRD pattern of bulk MoSe_2_ and MoSe_2_ NDs. Figure S2. FTIR spectra of FA-MoSe_2_@BSA NSs. (DOCX 145 kb)

## Abbreviations

BSA: Bovine serum albumin
FA: Folic acid
MoSe_2_: Molybdenum selenide
NDs: Nanodots
NIR: Near infrared
NSs: Nanospheres
PEG: Polyethylene glycol
PTT: Photothermal therapy
RT: Radiotherapy
