# Supplementary material for: Tumor-Targeted and Biocompatible MoSe2 Nanodots@Albumin Nanospheres as a Dual-Modality Therapy Agent for Synergistic Photothermal Radiotherapy
Source: Nanoscale Res Lett. 2019 Feb 26;14:67. doi: 10.1186/s11671-019-2896-z (PMC6391510; doi:10.1186/s11671-019-2896-z)
Supplement: Supplementary file 1 — Figure S1. XRD pattern of bulk MoSe2 and MoSe2 NDs. Figure S2. FTIR spectra of FA-MoSe2@BSA NSs. (DOCX 145 kb) [file 11671_2019_2896_MOESM1_ESM.docx]

**Additional file 1**

**Supplementary Material**

**Tumor targeted and biocompatible MoSe_2_ nanodots@albumin nanospheres as a dual-modality therapy agent for synergistic photothermal radiotherapy**

**Feng Qi ^1,^*, Ruizhen Liu^1^**

^1^ Department of Radiotherapy, First People's Hospital of Shangqiu City, Shangqiu 476100, China.

*Correspondence: Feng Qi, Email: [fengqi_radio@foxmail.com](mailto:fengqi_radio@foxmail.com); Tel: +86-13643708069

Ruizhen Liu: [docliuholy@foxmail.com](mailto:docliuholy@foxmail.com)





**Figure S1**. XRD pattern of bulk MoSe_2_ and MoSe_2_ NDs.





**Figure S2**. FTIR spectra of FA-MoSe_2_@BSA NSs.

Fig. 2a shows the XRD pattern of MoSe2 NDs. The
diffraction peak at 13.4° belongs to the (002) plane, matching
the peak position of 2H-MoSe2 (JCPDS, 87-2419). The distinct
(002) peak indicates the existence of few-layers in the c-axis of
MoSe2 NDs. The diffraction peak of (100) plane for MoSe2
NDs broadens significantly in comparison with bulk MoSe2,
which may originate from the size reduction of MoSe2 NDs
